# Distribution and Association of Cardiometabolic Risk Factors Among Youth From Al Ain City, United Arab Emirates

**DOI:** 10.7759/cureus.48681

**Published:** 2023-11-12

**Authors:** Charu Sharma, Abubaker Hassan, Sania Al Hamad, Javed Yasin, Juma Alkaabi, Elhadi H Aburawi

**Affiliations:** 1 Department of Internal Medicine, College of Medicine and Health Sciences (CMHS) United Arab Emirates University, Al Ain, ARE; 2 Public Health, United Arab Emirates University, Al Ain, ARE; 3 Pediatrics, College of Medicine and Health Sciences (CMHS) United Arab Emirates University, Al Ain, ARE; 4 Pediatrics/Pediatric Cardiology, College of Medicine and Health Sciences (CMHS) United Arab Emirates University, Al Ain, ARE

**Keywords:** uae, inflammation and heart failure, deranged lipid profile, extreme obesity, youth, cardiometabolic risk factors

## Abstract

Aim: The purpose of this study was to examine the distribution of cardiometabolic risk factors (CMRF) among UAE University students.

Methods: The present study employed a cross-sectional design to investigate the characteristics of a sample of young individuals aged 17-26 years. The participants were exclusively drawn from the student population of UAE University. Anthropometric measurements, including weight, height, blood pressure, and random blood collection, were conducted. The statistical methods employed for comparison included the Chi-square test, Fisher's exact test, and either the two-sample t-test or the Wilcoxon rank sum test. Logistic models, both adjusted and unadjusted, were utilized to evaluate the correlation between excessive body weight and various cardiovascular and metabolic risk factors (CMRFs). All P-values were calculated using a two-sided test, and a significance level of P < 0.05 was used to determine statistical significance. The statistical computing and graphics software R (version 4.2.2) was utilized to perform all data analyses.

Results: Among the 269 individuals who took part in the study, a significant proportion of 55% (n=148) were identified as males. Additionally, 36% (n=97) of the participants reported having a family history of hypertension. It is worth noting that the total sample consisted of younger individuals, with a mean age of 19 years (standard deviation ±1.8). There was a significant association between overweight/obesity and male gender (p=0.003), as well as having a family history of heart attack (p=0.038), high lipid profile, and high-sensitivity C-reactive protein (hs-CRP). There was no observed correlation between a family history of hypertension and HbA1C levels in individuals with a non-normal weight. substantially elevated cardiometabolic risk variables, including systolic blood pressure (SBP) equal to or greater than 130 mmHg, diastolic blood pressure (DBP) equal to or greater than 80 mmHg, triglyceride (TG) levels equal to or greater than 150 mg/dL, high-density lipoprotein cholesterol (HDL-C) levels equal to or less than 35 mg/dL, apolipoprotein B (Apo B) levels equal to or greater than 1.3 g/L, and high-sensitivity C-reactive protein (hs-CRP) levels equal to or more than 1 mg/L, were observed to be substantially more prevalent in individuals with excess body weight compared to those with normal weight. Furthermore, the likelihood of having low HDL levels is observed to increase by 14% (Adjusted Odds Ratio = 1.14, 95% Confidence Interval [1.07 to 1.23]) among students who have extra body weight, while accounting for age and gender as controlling factors.

Conclusions: Excess body weight, already in youth, was associated with increased CMRF, particularly high SBP and TG plus low HDL-C.

## Introduction

In the Middle Eastern (ME) population, cardiovascular diseases (CVD) in general and, specifically, acute myocardial infarction occur in younger patients with higher CVD risk factors (97%) than the population of the rest of the world (90.4%) [1]. The age-adjusted prevalence rate for CVD worldwide in 2020 was 7354.1 per 100,000, while in the ME, it was 10148 per 100,000 [2]. Countries in the ME region are classified mainly as high- and middle-income countries. There has been a rapid transformation of the civilizations in the ME countries from rural to urban lifestyles over the last six or seven decades. Despite the decrease in CVD mortality in many developed countries in the last three decades, the rates have risen to about 80% in ME countries [3,4]. The prevalence of obesity among adults is rapidly increasing in the developing world, including the Gulf region [5]. Moreover, cardiometabolic risk factors (CMRFs) are more prevalent among children and youth who are overweight or obese than those with a healthy weight [6,7]. Previous studies have affirmed that the prevalence of cardiovascular risk factors in childhood has increased, and biomarkers of adverse cardiovascular outcomes have already been found in childhood with obesity [8]. Severe obesity increases carotid artery wall stiffness and is associated with endothelial dysfunction, an early marker of atherosclerosis, as early as in childhood [9-11]. Recent studies have also reported that excess body weight in adolescents has important implications for developing CVD and diabetes [7,12,13].

In our recently published study [7], we reported that the prevalence of CMRF in children, such as high levels of triglycerides, total cholesterol, and glucose, was quite high in Emirati school-aged children with excess body weight. This may be because of the consumption of unhealthy food, a sedentary lifestyle, and family history. Identifying abnormal weight gain early and prioritizing the primary prevention of CVD in children, adolescents, and, by default, youth will reduce the risks of undesirable structural and metabolic changes.

Therefore, this study aimed to observe the distribution of CMRF among UAE university students along with the sex-based distribution pattern to identify the factors associated with increased CMRF. Furthermore, we plan to conduct a follow-up of this cohort with related measures of obesity, with the possibility of intervention to identify the long-term outcomes and help understand the evolution of CMRF and the future development of CVD.

## Materials and methods

Study design

This study was conducted in the Al Ain region of the Abu Dhabi Emirates between 2019 and 2020 among UAE nationals, mainly students aged 17-26 years enrolled at the UAE University (UAEU).

Study subjects

An official email invitation was sent by the Assistant Dean for Students Affairs, CMHS, to 700 UAEU students aged between 17 and 26 years. The response rate was 38%. All the volunteers were informed about the study, and those who agreed to participate were required to sign the consent form.

Anthropometric measurements

All the students who consented to participate in the study underwent anthropometric measurements, including weight, height, and waist circumference, by a trained nurse. Weight and height were measured using the stadiometer (a portable digital scale), wherein the students were instructed to assume an upright posture, aligning their heads, backs, and buttocks in a vertical orientation with respect to the height gauge. The height measurement was taken next and was rounded off to the nearest 0.5 cm. We used unbent tapes to measure the circumference of the waist, starting at the point that was halfway between the base of the rib cage and the highest point of the iliac crest. The body mass index (BMI) was calculated using the following formula: BMI = weight (kg)/height (in m).

The participants were classified into two groups based on National Institutes of Health (NIH) criteria: normal weight (BMI ≤ 24.9 kg/m2) and overweight plus obese (BMI≥25 kg/m2) [14-16]. The measurement of blood pressure (BP) was conducted utilizing a meticulously calibrated Omron M6 IntelliSense automatic BP monitor (Healthcare, Kyoto, Japan). The sleeve cuffs used during the measurement were appropriately selected to accommodate the size of each individual’s arm.

Clinical variables

Blood samples were collected from all the participants by venipuncture and stored in thermal boxes. The plasma was separated from whole blood, divided into aliquots, and stored at −80ºC until further use. Blood glucose, hemoglobin A1c (HbA1c), total cholesterol (TC), LDL-C, HDL-C, triglycerides (TG), apolipoprotein A (Apo A), and Apo B were estimated in all biological samples on an automated analyzer, the Integra 400 Plus (Roche Diagnostics, Mannheim, Germany).

The Beckman Coulter Synchron Clinical System (UniCel DxC-800) was used to measure high-sensitive C-reactive protein (hsCRP). The laboratory also checked internal quality controls before running samples and participated in the College of American Pathologists Proficiency Testing External Quality Assurance program. We examined gamma-glutamyl transferase (GGT), a biomarker for fatty liver, using an Integra 400 Plus automated analyzer (Roche Diagnostics).

Description of risk factors

Normal and abnormal body weight were based on BMI and classified into the above-mentioned two groups.

Standardized age-specific cut-off values were used to define the abnormal values for TC: ≥200 mg/dL, HDL-C: <35 mg/dL, LDL-C: ≥130 mg/dL, TG: ≥150 mg/dL, systolic BP (SBP), and diastolic BP (DBP): ≥95th percentile or ≥130/80 mmHg, HbA1c: >5.7%, and blood glucose: ≥100 mg/dL.

Statistical analysis

In this study, all the statistical analyses were conducted using R (version 4.2.2) for computing graphics. The categorical variables were analyzed as frequencies and percentages, while continuous variables were summarized using mean (±SD) or median (interquartile range [Q25, Q75]). The chi-squared or Fisher’s exact test (categorical variables) and the two-sample t-test or Wilcoxon rank sum test (continuous variables) were used to compare sociodemographic and clinical characteristics between BMI categories. Crude and adjusted logistic models were fitted to assess the association between excess body weight and different CMRFs. For all comparisons, P <0.05 was considered to indicate statistically significant differences.

Ethics approval

The study protocol received approval from the Human Ethics Committee in the year 2020 from the College of Medicine and Health Sciences, United Arab Emirates University (ERH-2020-6058 2020-01), located in Al Ain, UAE. Written consent was obtained before the initiation of the study from all participants. The objectives and outcome of the study were explained, and patient anonymity was preserved by not disclosing personal information.

## Results

Results

**Figure 1:**
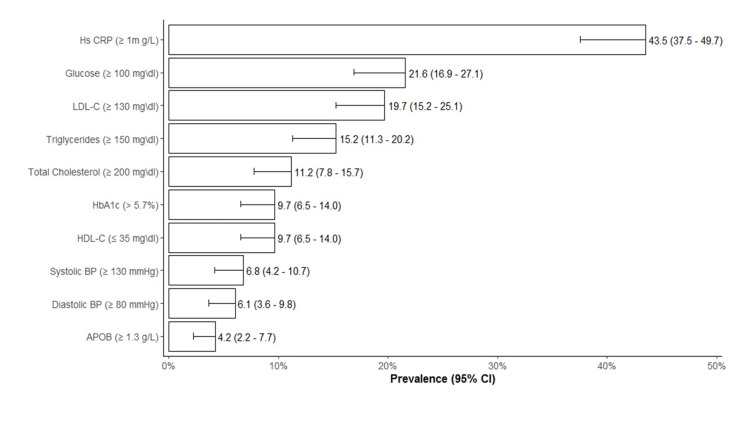
Prevalence and 95% confidence interval of cardiometabolic risk factors in youth.

Table 1 shows the sociodemographic and clinical characteristics of the study participants. There were 269 participants (mean age: 19±1.8 years), of whom 55% (n = 148) were male and 36% (n = 97) had a history of hypertension. The prevalence of excess body weight was 42% (95% confidence interval [CI]: 36.1-48.1%). hs-CRP (≥ 1 mg/L) was the most prevalent CMRF and was reported in 43.5% (95%CI: 37.5-49.7) of the study sample. Blood glucose (≥ 100 mg/dL) and LDL-C (≥ 130 mg/dL) were presented in 21.6% (95%CI, 16.9-27.1) and 19.7% (95%CI: 15.2-25.1), respectively. Only 4.2% (95% CI: 2.2-7.1) of participants were presented with Apo B (≥ 1.3 g/L) (Figure 1).

Overweight/obesity was significantly associated with being male (P = 0.003), having a family history of heart attack (P = 0.038), and having high levels of lipid profile and hs-CRP. We found no association between the presence of a family history of hypertension and HbA1C with abnormal weight (Table 1). 

**Table 1:** Sociodemographic and clinical characteristics of the study population by BMI category (N = 269). ^1^ Mean (±SD); median (Q1, Q3); n (%) ^2^ Two Sample t-test; Wilcoxon rank sum test; Pearson’s chi-squared test; Fisher’s exact test HDL-C: high-density lipoprotein; LDL-C: low-density lipoprotein; TG; Triglycerides; Apolipoprotein A (Apo-A); Apolipoprotein B (Apo-B), hemoglobin A1c (HbA1c),  gamma-glutamyl transferase (GGT), hs-CRP: high-sensitivity C-reactive protein

Variable	Overall N = 269^1^	Normal Body Weight N = 156^1^	Excess Body Weight N = 113^1^	P-value^2^
Age (years)	19.64 (±1.77)	19.68 (±1.70)	19.58 (±1.86)	0.600
Sex				0.003
Female	121 (45%)	82 (53%)	39 (35%)	
Male	148 (55%)	74 (47%)	74 (65%)	
BMI (kg/m^2^)	24 (21, 29)	22 (20, 23)	30 (27, 34)	<0.001
Height/Waist Circumference Ratio	2.10 (±0.42)	2.31 (±0.39)	1.82 (±0.27)	<0.001
Family history of HTN	97 (36%)	49 (31%)	48 (42%)	0.062
Family history of DM	88 (33%)	45 (29%)	43 (38%)	0.11
Family history heart attack	9 (3.3%)	2 (1.3%)	7 (6.2%)	0.038
SBP (mmHg)	115 (±11)	112 ± (10)	120 (±11)	<0.001
DBP (mmHg)	69.7 (±6.0)	68.6 (±5.6)	71.3 (±6.2)	<0.001
Total cholesterol (mg\dL)	164 (±30)	160 (±30)	169 (±30)	0.016
HDL-C (mg\dL)	51 (±14)	54 (±14)	46 (±13)	<0.001
LDL-C (mg\dL)	105 (±29)	101 (±28)	111 (±30)	0.003
TG (mg\dL)	84 (57, 123)	69 (52, 108)	105 (77, 144)	<0.001
Apo A (g/L)	1.40 (±0.22)	1.43 (±0.22)	1.36 (±0.23)	0.008
Apo B (g/L)	0.82 (±0.23)	0.78 (±0.21)	0.88 (±0.24)	<0.001
Blood glucose (mg/dL)	86 (79, 96)	84 (78, 95)	87 (81, 100)	0.028
HbA1c (%)	5.09 (±0.53)	5.04 (±0.43)	5.16 (±0.64)	0.077
GGT (U/L)	19 (15, 25)	18 (13, 23)	21 (16, 27)	0.001
hs-CRP (mg/L)	0.85 (0.29, 1.87)	0.46 (0.26, 1.29)	1.26 (0.50, 2.68)	<0.001

**Figure 2:**
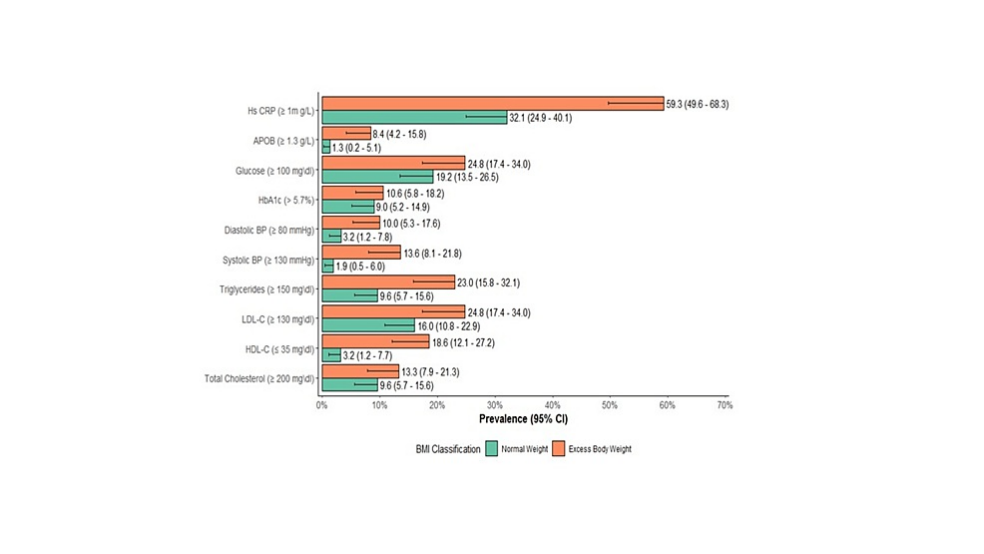
Prevalence and 95% confidence interval of cardiometabolic risk factors in youth by BMI classification.

Cardiometabolic risk factors such as SBP ≥130, DBP ≥80 mmHg, TG ≥150, HDL-C ≤35, Apo B ≥1.3 g\L, and hs-CRP ≥1 mg/L were significantly higher in individuals with excess body weight than those with normal weight. HbA1c >5.7%, TC ≥200 mg/dL, and LDL-C ≥130 mg/dL were higher in the obese group; however, these findings were not statistically significant (Figure 2).

Male subjects were significantly younger than female subjects (19.11±1.57 years vs. 20.28±1.79 years; P < 0.001). Moreover, 50% of the male participants had excess body weight and significantly higher BMI (P < 0.001); and total cholesterol (P < 0.001), LDL-C (P = 0.001), and triglyceride (P < 0.001) levels.

There was no significant difference between male and female subjects with regard to family history of HTN, DM, or heart attack. Female subjects had significantly lower Apo B (P < 0.001), blood glucose (P < 0.001), HbA1c (P < 0.001), and GGT (P < 0.001) than male subjects (Table 2).

**Table 2:** Sociodemographic and clinical characteristics of the study population by sex (N = 269). ^1^ Mean (±SD); Median (Q1, Q3); n (%) ^2^ Two Sample t-test; Wilcoxon rank sum test; Pearson’s chi-squared test; Fisher’s exact test HDL-C: high-density lipoprotein; LDL-C: low-density lipoprotein; TG; Triglycerides; Apolipoprotein A (Apo-A); Apolipoprotein B (Apo-B), hemoglobin A1c (HbA1c), gamma-glutamyl transferase (GGT), hs-CRP: high-sensitivity C-reactive protein

Variable	Female N = 121^1^	Male N = 148^1^	P-value^2^
Age (years)	20.28 (±1.79)	19.11 (±1.57)	<0.001
BMI classification			0.003
Normal weight	82 (68%)	74 (50%)	
Excess body weight	39 (32%)	74 (50%)	
BMI (kg/m^2^)	23 (21, 26)	25 (22, 31)	<0.001
Height/waist circumference ratio	0.44 (±0.07)	0.54 (±0.09)	<0.001
Family history HTN	47 (39%)	50 (34%)	0.400
Family history DM	45 (37%)	43 (29%)	0.200
Family history heart attack	7 (5.8%)	2 (1.4%)	0.083
SBP (mmHg)	108 (±8)	121 (±9)	<0.001
DBP (mmHg)	71.1 (±5.4)	68.6 (±6.2)	<0.001
Total cholesterol (mg\dL)	157 (±24)	169 (±33)	<0.001
HDL-C (mg\dL)	57 (±15)	46 (±11)	<0.001
LDL-C (mg\dL)	99 (±24)	110 (±32)	0.001
TG (mg\dL)	66 (52, 93)	107 (72, 144)	<0.001
Apo A (g/L)	1.41 (±0.23)	1.40 (±0.22)	0.600
Apo B (g/L)	0.74 (±0.18)	0.89 (±0.24)	<0.001
Blood glucose (mg/dL)	83 (78, 90)	87 (80, 104)	<0.001
HbA1c (%)	4.95 (±0.35)	5.21 (±0.62)	<0.001
GGT (U/L)	14 (12, 17)	23 (19, 28)	<0.001
hs-CRP (mg/L)	0.87 (0.27, 1.93)	0.85 (0.31, 1.77)	0.9

Table 3 shows the results of the logistic regression analysis that was performed to study the association of excess body weight on the crude likelihood of different CMRFs adjusted for age and sex. Overall, excess body weight was independently associated with an increased risk for the studied CMRFs; moreover, the adjusted ORs were slightly attenuated compared to crude estimates. The unadjusted logistic regression analysis indicated that the likelihood of high hs-CRP (crude odds ratio; cOR = 1.31, [95%CI: 1.17-1.47], P < 0.001), low HDL-C (cOR = 1.17, [1.09-1.25], P < 0.001), and high systolic blood pressure (cOR = 1.12, [1.06-1.19], P = 0.020) were statistically associated with obese adolescents. After controlling for age and sex, the odds of having high hs-CRP, low HDL, and high SBP significantly increased by 34% (adjusted odds ratio; aOR = 1.34, [1.19-1.50], P < 0.001); 14% (aOR = 1.14, [1.07-1.23], P < 0.001); and 11% (aOR = 1.11, [1.04-1.17], P < 0.001), respectively, in youth with excess body weight. 

**Table 3:** Association between excess body weight and different cardiometabolic risk factors using crude and adjusted logistic regression analysis. OR: odds ratio; CI: confidence interval. 1 Logistic regression adjusted for age and sex. HDL-C: high-density lipoprotein; LDL-C: low-density lipoprotein; TG; Triglycerides; Apolipoprotein A (Apo-A); Apolipoprotein B (Apo-B), hemoglobin A1c (HbA1c), gamma-glutamyl transferase (GGT), hs-CRP: high-sensitivity C-reactive protein

Risk Factor	Crude OR (95% CI)	P-value	Adjusted OR (95% CI)^1^	P-value
SBP ≥130 mmHg				
Normal weight	Ref	<0.001	Ref	<0.001
Excess body weight	1.12 (1.06–1.19)		1.11 (1.04–1.17)	
DBP ≥80 mmHg				
Normal weight	Ref	0.02	Ref	<0.04
Excess body weight	1.07 (1.01–1.13)		1.06 (1.00–1.13)	
TG ≥150 mg\dL				
Normal weight	Ref	<0.001	Ref	0.02
Excess body weight	1.14 (1.05–1.25)		1.11 (1.02–1.21)	
HDL-C ≤35 mg\dL				
Normal weight	Ref	<0.001	Ref	<0.001
Excess body weight	1.17 (1.09–1.25)		1.14 (1.07–1.23)	
Apo B ≥1.3 g\L				
Normal weight	Ref	<0.001	Ref	0.02
Excess body weight	1.07 (1.03–1.13)		1.06 (1.01–1.11)	
hs-CRP ≥1 mg/L				
Normal weight	Ref	<0.001	Ref	<0.001
Excess body weight	1.31 (1.17–1.47)		1.34 (1.19–1.50)	

## Discussion

This study reported that excess body weight among UAEU students can lead to high CMRFs. The distribution of fat in the body plays an important role in endothelial dysfunction. The excess body fat is reported to enhance atherogenic risk related to CMRFs, leading to arteriosclerosis development primarily among children and adolescents [17]. Diseases like obesity and diabetes may exacerbate the development of atherosclerosis as they affect early life [18,19]. Atherogenesis is frequently linked to dyslipidemia, a significant risk factor for the development of atherosclerosis and cardiovascular disorders [20].

Atherogenic dyslipidemia is a metabolic abnormality that is considered a defining characteristic of the metabolic syndrome (MetS). It is characterized by elevated levels of TGs, increased very low-density lipoprotein (VLDL) and LDL, and decreased HDL levels [21]. The occurrence of AO (abdominal obesity) exhibits a robust correlation with atherogenic dyslipidemia in the younger population. Multiple studies have demonstrated a correlation between AO and an atypical lipid profile in the pediatric and adolescent populations. This correlation is particularly evident in relation to elevated levels of low-density lipoprotein cholesterol (LDL-C), reduced levels of high-density lipoprotein cholesterol (HDL-C), and the presence of hyperglycemia across all age groups [22].

Within the population of the UAE, there exists a complex interplay between various risk factors associated with cardiovascular disease (CVD), and the subsequent accumulation of these factors has been reported earlier between 2016 and 2018 in young and middle-aged adults (18-40 years) in the UAE. The study revealed a high prevalence of CMRFs, which exhibit significant associations among themselves, thereby contributing to a substantial burden of risk factors for CVD. Overall, obesity had the strongest relationship with all metabolic abnormalities [23].

CMRF burden and distribution in relation to sociodemographic and behavioral variables were then examined in the same cohort. The study found 26.5% obese, 11.7% dysglycemic, 62.7% dyslipidemic, and 2.4% hypertensive participants; furthermore, 22.5% had central obesity. According to the findings of the study, CMRFs are extremely common among the young Emirati individuals who participated in the UAE healthy future project. The disparity in the CMRFs’ distribution across different social and behavioral groups might be considered to target preventative interventions that are group-specific [24].

Another study that collected data from December 2012 to May 2013 in the northern UAE region among adult participants (mean age: 42.8 years) showed a higher incidence of dyslipidemia. Sex, age, smoking, central obesity, and diabetes were predictors of dyslipidemia [25].

This study is different from other reported studies on the UAE population in many aspects, mainly age, homogeneities, and the inflammatory status of the population. This study was conducted in 2020 among UAEU students, where the population is homogenous. All the students were aged between 17 and 25 years, which is classified as “youth” per the WHO criteria. We extensively studied the inflammation predicted by levels of hs-CRP, Apo A and B, and liver injury markers (GGT).

In a multicentric and school-based study conducted among Brazilian adolescents, the presence of excessive body weight was shown to potentially contribute to the emergence of various CMRFs. The prevalence of this condition was determined through the assessment of aberrant glucose levels, dyslipidemia, and hypertension, revealing a higher occurrence among adolescents with obesity than those with a healthy body weight. Additionally, it exhibits a propensity for persistence and augmentation of the susceptibility to early cardiovascular disease (CVD) during the adult lifespan, thereby potentially elevating the likelihood of developing coronary heart disease, stroke, diabetes mellitus, and overall mortality in the adult stage of life [26].

Anthropometric measures of body fat like waist circumference, waist-to-hip ratio, and waist-to-height ratio can predict abdominal obesity (AO). In the present study, we observed that the ratio of waist-to-height circumference was significantly different among the two groups (p < 0.001). Several studies have found that surrogate AO indicators are independent risk factors for T2DM, dyslipidemia, hypertension, and coronary artery disease [27].

The primary therapeutic interventions for adolescents afflicted with severe obesity primarily revolve around implementing lifestyle adjustments, encompassing the adoption of a nourishing dietary regimen and the augmentation of physical exertion. Nonetheless, this particular intervention elicits discreet alterations in weight over a limited duration, with individuals typically persisting within the confines of their original BMI classification [28]. Additional therapeutic interventions for obesity in adolescents encompass pharmacological agents and bariatric surgical procedures [29]. Despite its efficacy in promoting weight loss and enhancing cardiometabolic risk factors, the enduring consequences of this particular intervention on the morbidity and mortality rates among adolescents remain elusive because of the limited duration of follow-up observed in prior investigations. Given the intricate nature of managing obesity in adolescents across various classifications, it becomes imperative to initiate preventive measures during childhood. This entails exploring novel avenues to facilitate the alteration of detrimental behaviors. Alternatively, interventions can be implemented during intrauterine life to create a conducive environment for optimal fetal development [27].

Our adjusted models showed that CMRF prevalence increases with weight excess severity. Obesity-related CMRFs vary in high-income countries. A Korean study of 1326 adolescents found that metabolic risk factors increase with obesity. Thus, severely obese teenagers have greater metabolic risk factors than those with less obesity [30].

Another study on severely obese children and young adults reported the association with an increased prevalence of CMRFs, particularly among boys and young men. The study also reported that the prevalence of these abnormal values appears to be dependent on both age and the severity of obesity. In that study, some CMRFs showed significant differences related to obesity, while others did not [8]. The observed variations could potentially be elucidated by the disparate thresholds employed to define abnormal values in the respective investigations. The observed phenomenon may also be linked to distinct dietary patterns and variations in sugar consumption across different regions. For instance, countries in the United States exhibit the highest levels of sugar-sweetened beverage consumption, whereas those in Asia display the lowest levels [31].

Limitations

The major limitation of this study is that it is based on opportunistic recruitment of participants. This inclusion may add to the selection bias and potentially affect the findings of the study. The study should be broadened to different emirates of the UAE and also to have participants of various ethnicities. Another limitation is that we didn't explore more cardiometabolic risk assessment factors, which could more strongly support the findings of the study. 

## Conclusions

The prevalence of cardiometabolic risk factors is high in this cohort. Excess body weight in youth is associated with increased cardiometabolic risk factors: high systolic blood pressure and triglycerides and low-density lipoprotein cholesterol. Combating obesity and its cardiometabolic risk factors should be started as early as childhood, both at home and in school. This will prevent, or at least delay, the irreversible damage of the atherosclerosis process before it becomes too late.
